# Clinical pictures, treatments, and resource use of norovirus gastroenteritis in long-term care facilities: a survey with a chart review in Japan

**DOI:** 10.1186/s12877-020-01549-0

**Published:** 2020-04-21

**Authors:** Saori Fujiki, Tatsuro Ishizaki, Takeo Nakayama

**Affiliations:** 1 Department of Health Informatics, Kyoto University School of Public Health, Yoshida Konoe-cho, Sakyo-ku, Kyoto, 606-8501 Japan; 2 Human Care Research Team, Tokyo Metropolitan Institute of Gerontology, 35-2 Sakae-cho, Itabashi-ku, Tokyo, 173-0015 Japan; 3 Department of Health Informatics, Kyoto University School of Public Health, Yoshida Konoe-cho, Sakyo-ku, Kyoto, 606-8501 Japan

**Keywords:** Resource use, Norovirus gastroenteritis, Long-term care facilities, Chart review, Japan

## Abstract

**Background:**

Outbreaks of norovirus infection can have detrimental impacts on long-term care facilities. This study investigated the incidence, clinical picture, treatment and resource use of norovirus gastroenteritis in long-term care facilities.

**Methods:**

Nineteen facilities in Osaka and Kyoto, Japan participated in questionnaire surveys conducted between 2009 and 2011 regarding the incidence of norovirus gastroenteritis. From clinical charts, the characteristics, symptoms, and treatment of infected residents were analyzed. Total drug cost per infected resident was calculated by multiplying the unit price for each drug by the daily dose and the number of days administered and summing the costs for each drug (USD 1 = JPY 100).

**Results:**

Over the 3-year period, 8 outbreaks of norovirus gastroenteritis occurred in 6 facilities. The mean clinical course of 107 infected residents in five facilities that granted permission to examine patients’ medical records was 4 days, with all but one resident presenting with vomiting and/or diarrhea, and 84 (79%) also presenting with associated symptoms. Of 107 infected residents, 72 (67%) were isolated. The proportion of infected residents isolated varied from 50 to 100% depending on the facility. Of the infected residents, 81 (76%) received some type of medication, the most common being infusion (67 patients, 63%) and antibiotics (30 patients, 28%). The median total cost of all drugs administered was USD 4.4, and the median drug cost per infected resident per day was USD 2.0. Total drug cost over the entire treatment period was the highest for antibiotics, at USD 8.6.

**Conclusion:**

Clinical course was similar to those of norovirus cases examined at other long-term care facilities. The majority of infected residents received some type of medication. Although the economic burden was not large, not a few infected residents were given antibiotics, which are ineffective for norovirus.

## Background

Norovirus gastroenteritis (NG) is one of the infectious diseases of greatest concern for elderly residents of long-term care facilities (LTCFs). Following an incubation period of 12 to 48 h, patients with NG present with primary symptoms of nausea or vomiting and diarrhea [1]. Although the majority of infected individuals recover within 3 days, NG can be fatal in elderly persons. The risk of fatality in elderly persons is estimated to be approximately 20 times that in young people [1, 2]. In the United States, there are approximately 20 million cases of acute gastroenteritis each year, with the majority occurring in LTCFs [1, 3]. In Japan, the number of patients infected with NG each year ranges between 10,000 and 15,000. Cases first appear and start to increase in number in October, peaking in January of the following year and decreasing in March [4]. In Japan, NG outbreaks occur most frequently in nurseries, restaurants, and facilities [5].

Every year, the Japanese government issues announcements regarding the prevention of NG. The Infection Control Manual in a Facility for the Elderly published by the Ministry of Health, Labour and Welfare (MHLW) contains recommendations that patients with similar symptoms should be placed in the same room, and that patients be supplemented with an infusion when necessary. The manual also describes the method for disinfecting tableware and clothing polluted with vomitus, the method for disposing of vomitus and excreta, and the criteria to stop acute responses against the infection expanding. LTCFs create their own manuals based on the MHLW manual and try to deal with NG based on them. More than 80% of facilities have infection prevention manuals, have taken steps to educate and ensure that their staff are aware of the content of these manuals, and have established infectious disease response committees [6, 7]. The economic costs to LTCFs of an NG outbreak, including losses associated with suboptimal occupancy rates, care of infected patients, sick leave and overtime of staff, cleaning fees, etc., can be substantial [8–11].

In many cases, NG diagnoses in facilities are made comprehensively based on the clinical course and the state of infection of other residents without the use of a diagnostic kit. There is no specific treatment for NG; treatment consists simply of supportively providing fluid and nutritional supplementation [12]. However, because there is no official scheme in Japan for evaluating or monitoring the care of facility residents, the actual state of care is not known. Although reports on the total number of patients, clinical course, infection source and path, and genetic characteristics of causal agents from the outbreak to the end of NG in facilities exist, few studies in Japan and other Asian countries have also included examination of medical records [13, 14]. Furthermore, there have been no studies on medical resource use by facilities with respect to NG.

This study aimed to clarify the incidence, clinical picture, treatment, and medical resource useassociated with NG in LTCFs in Japan.

## Methods

Only a brief description of the sample and methods, which are described in detail elsewhere, is provided [15]. A two-stage survey (Surveys I and II) was performed. For Survey I, letters were written in October, 2011 to facilities belonging to the Osaka and Kyoto branches of the Japan Association of Geriatric Health Service Facilities (JAGHSF) requesting their participation in this study. JAGHSF is an incorporated association whose members are geriatric intermediate care facilities. Geriatric intermediate care facilities are inpatient facilities that provide long-term care such as daily life support and rehabilitation under medical supervision to elderly patients with stable medical conditions that do not require hospitalization but do require nursing care, with the goal that the patients will eventually return home [16–18]. In Survey I, information was obtained regarding NG outbreaks in two periods, from October, 2009 to March, 2010 (2009 survey) and from October, 2010 to March, 2011 (2010 survey). In Survey II, the same information was obtained for October, 2011 to March, 2012 (2011 survey). The present study analyzed the data from the 19 facilities that participated in all three surveys. These participating facilities were of average size among comparable ones, with a mean occupancy rate of 97.0% (range: 85.0 to 116.0) and a mean capacity of 95.2 (range: 50 to 120) residents.

The items investigated were NG incidence and restriction of new admissions due to the outbreak (duration of restriction, number of new admissions to the facility during the restricted period), refer to Additional files 1 and 2. Residents who were suspected of having NG and were treated accordingly by facilities were designated “infected residents.” Definition of outbreak is an epidemic limited to localized increase in the incidence of a disease [19]. If an NG outbreak had occurred, the facility in question was visited with permission, and the medical records of infected residents were examined to collect the following information: sex, age, nursing care level, symptoms, symptom duration, use (or non-use) of an NG diagnostic kit, isolation circumstances, treatment during the symptomatic period (name of medications, daily dose, and number of days administered for each medicine), and outcomes. Symptoms were classified into primary symptoms (vomiting, diarrhea) and associated symptoms (symptoms other than primary symptoms such as decreased appetite, fever of 37 °C or greater, general malaise). In cases where permission to examine medical records was not granted, the facility was asked to provide the above information for infected residents. The symptom durations were defined as the existence of symptom during the stay in the facility regardless of the infected residents’ outcomes. Outcome data of patients who were transferred and hospitalized were confirmed by the facilities’ records. Statistical analyses were performed comparing the group of residents who received treatment and the group of residents who did not receive treatment for the following items: age, care level, symptom duration, number of isolated residents, isolation duration, presentation of primary symptoms, and outcome (recovery or death). Independent *t*-tests were performed for continuous independent variables, and chi-squared tests were performed for categorical independent variables. IBM SPSS Ver. 24 was used to perform all analyses.

The total drug cost per infected resident was calculated by multiplying the unit price for each drug by the daily dose and the number of days administered and summing the costs for each drug. For drug prices, the National Health Insurance Drug Price Standard from April, 2012 was used. When a price was not available, price information was obtained from the facility administering the drug. For the cost of infusion treatment, referring to a medical service points list, the daily price for the procedure and materials necessary for infusion treatment such as needle, route, tape, cotton (USD 10) was multiplied by the number of days administered, and the price of one plastic cannula-type intravenous catheter (USD 1) was added per patient. For the cost per day of each drug administered, the median cost per day for all patients receiving that drug was used. For the total treatment cost per patient per day, the total cost of treatment for each patient was divided by the number of days that treatment was administered. Costs were estimated based on an assumed exchange rate of USD 1 = JPY 100.

## Results

### NG outbreaks

Between 2009 and 2011, there were 8 outbreaks: 3 in 2009, 3 in 2010, and 2 in 2011. Between 2009 and 2011, NG outbreaks occurred in 6 of the 19 facilities (32%), with outbreaks occurring in two consecutive years in 2 of the facilities. In all 6 facilities where NG outbreaks occurred, NG prevention measures were implemented the year when the outbreaks occurred. These facilities had a mean capacity of 102 (range: 95 to 120) residents, a median average occupancy rate of 98% (25th percentile, 95%; 75th percentile, 104%), with mean nursing care levels similar to those of all 19 facilities. Of 8 outbreaks, 7 occurred from December to January of the following year and 1 outbreak occurred in February. Six of the 8 outbreaks resulted in restricting the acceptance of new residents, with a median restriction period of 20 days (25th percentile, 13 days; 75th percentile, 38 days); the duration of restriction was not known in one of six outbreaks.

### Infected residents

The number of infected residents in each facility is shown in Table 1. From 2009 to 2011, there were 146 infected residents in 6 facilities where NG outbreaks occurred. In the 5 facilities (Facilities A to E) that granted permission to examine patients’ medical records, the clinical course and treatment were investigated for 107 patients (A: 4, B: 41, C: 2, D: 24, E: 36). Of these 107 individuals, 21 (20%) were men, and 86 (81%) were women, with a mean age of 85.4 (range: 68 to 101) years, 42 (39%) could communicate but required assistance in carrying out some activities of daily living (ADL), 58 (54%) had difficulty communicating and required assistance in carrying out all ADL, and 7 (7%) could not communicate and required assistance in carrying out all ADL. NG was diagnosed using a diagnostic kit in 32 (30%) of the patients and on the basis of symptoms only in 75 (70%) of the patients. Among 32 patients diagnosed using a diagnostic kit, 17(53%) had positive.

**Table 1:** Facilities with norovirus infection outbreaks and the number of residents with norovirus gastroenteritis and isolation at each facility (2009–2011)

Year	2009	2010	2011	Total number in 2009–2011	Permission to review charts	Number of infected residents isolated (%^a^)
Facility A	4	0	0	4	yes	2 (50.0)
Facility B	41	0	0	41	yes	22 (53.7)
Facility C	0	2	0	2	yes	2 (100.0)
Facility D	0	2	22	24	yes	24 (100.0)
Facility E	0	0	36	36	yes	22 (61.1)
Facility F	20	19	0	39	no	.
Total number of residents with norovirus gastroenteritis				146	yes: 107	72 (67.3)

^a^ % value is a ratio of the number of infected residents isolated to the number of infected residents

### Symptoms exhibited by infected residents

Of the 107 patients whose records were examined, 106 (99%) presented with vomiting or diarrhea (both vomiting and diarrhea 52 (49%), diarrhea only 37 (35%), vomiting only 17 (16%)). Associated symptoms of some kind were observed in 84 (79%) patients, with some patients presenting with multiple symptoms (decreased appetite 69 (65%), fever of 37.0 °C or greater 59 (55%), general malaise 47 (44%), and vomiting of blood 2 (2%)). Median symptom duration for the 107 patients was 4 days (25th percentile, 2 days; 75th percentile, 6 days).

### Isolation of infected residents

The number of isolated infected residents is shown in Table 1. The most common isolation method used was to place infected residents in the same room. However, in cases where same-room placement was deemed difficult due to room shortages, divider curtains around the beds were used to set off areas for infected residents. Of the 107 patients, 72 (67.3%) were isolated. Median isolation duration was 4 days (25th percentile, 3 days; 75th percentile, 5 days).

### Treatment of infected residents

Of the 107 patients, 81 (76%) received some sort of medication or transfusion. No substantial difference was observed in the characteristics of residents who received or did not receive medication or transfusion (Table 2). A higher proportion of residents receiving medical treatment was isolated than residents not receiving medical treatment (*p* = 0.008). No other differences were observed between the two groups of residents.

**Table 2:** Characteristics of residents receiving and not receiving treatment

Item	Residents receiving treatment (*n* = 81)	Residents not receiving treatment (*n* = 26)	*P* value
Age, y			0.246
mean [min–max]	85.5 [70–101]	83.9 [68–96]	
Care level^a^			0.117
median [25–75%]	3 [2–4]	2.5 [2–3]	
Symptom duration, days			0.205
median [25–75%]	4.0 [3.0–6.0]	2.0 [2.0–5.3]	
Isolated residents,			0.008 < 0.05
no. of residents (%)	60 (74.1)	12 (46.2)	
Isolation duration, days			0.707
median [25–75%]	4 [3.0–5.8]	4.5 [2.0–5.8]	
Primary symptoms, no. of residents (%)			0.332
Vomiting only	11 (13.6)	6 (23.1)	
Diarrhea only	26 (32.1)	11 (42.3)	
Vomiting + diarrhea	43 (53.1)	9 (34.6)	
Other	1 (1.2)	0 (0.0)	
Outcome, no. of residents (%)			0.414
Recovered in facility	76 (93.8)	23 (88.5)	
Died in facility	1(1.2)	0(0.0)	
Recovered in hospital	3(3.7)	3(11.5)	
Died in hospital	1(1.2)	0(0.0)	

^a^Care level: Level of nursing care needed as defined in Japan’s Long-term Care Insurance system. Individuals needing assistance in carrying out activities of daily living (ADL) are assigned a care level from 1 to 5 depending on their level of need. The higher the care level, the greater the care needed

Drugs were administered in one of three ways: intravenously, orally, or as a suppository. Ten (9%) patients administered as a suppository were administered only once during fever. The number of patients receiving drugs, treatment duration, and costs of each drug are presented in Table 3. Treatment was administered intravenously to 67 (63%) patients, orally to 47 (44%) patients, and both intravenously and orally to 33 (31%) patients. Median treatment durations for drugs administered intravenously and orally were 2 and 3 days, respectively. The drug types administered, in order of most to least common, were infusion (67 patients, 63%), antibiotics (30 patients, 28%), antidiarrheals (27 patients, 25%), antipyretic (19 patients, 17%), and antiemetics (17 patients, 16%). The antibiotics administered consisted of new quinolones (21 patients, 70%), macrolides (6 patients, 20%), tetracyclines (2 patients, 3%), penicillins (2 patients, 3%), cephems (2 patients, 3%), and aminoglycosides (1 patient, 1%), with 4 of the 30 patients (13%) being prescribed two types of antibiotics.

**Table 3:** Administration period and costs of each administered drug

	No. of patients *N* = 107 (%)	No. of days administered	Cost USD
		median [25th -75th percentile]	median [25th -75th percentile]
Receiving medications	81 (75.7)	2.0 [1.0–5.0]	4.4 [1.8–12.2]
Medication Method			
Intravenous	67 (62.6)	2.0 [1.0–3.0]	2.4 [1.6–7.1]
Oral	47 (43.9)	3.0 [1.0–5.0]	4.0 [0.2–8.6]
Administered drug (method for administration)			
Infusion (intravenous)	67 (62.6)	1.0 [1.0–3.0]	2.4 [1.6–7.1]
Antibiotics (intravenous or oral)	30 (28.3)	3.0 [2.0–5.0]	8.6 [5.6–15.4]
Antidiarrheal (oral)	27 (25.2)	1.0 [1.0–4.0]	0.4 [0.2–0.7]
Antipyretic (oral or suppository)	19 (17.8)	1.0 [1.0–1.0]	0.2 [0.05–0.2]
Antiemetic (intravenous or oral)	17 (15.9)	3.0 [1.0–5.5]	1.5 [0.6–3.5]
Antacid, laxative (oral)	5 (4.7)	4.0 [2.5–4.0]	0.7 [0.4–0.7]

The median cost of all drugs administered was USD 4.4, and the median cost of drugs per infected resident per day was USD 2.0. Median drug cost over the entire treatment duration was the highest for antibiotics, at USD 8.6.

### Outcomes

Of the 107 patients, 99 (93%) recovered in facility, 1 (1%) died in facility, and 7 (6%) were transferred to hospitals. Of the 7 patients transferred to hospitals, 1 died in hospital.

## Discussion

This retrospective medical record review showed the clinical pictures, treatment, and medical resource use related to NG outbreaks at 19 typical geriatric facilities in Japan. From 2009 to 2011, there were 8 NG outbreaks in 6 facilities (32%), resulting in 146 infected residents. Analysis of the medical records of 107 infected residents in 5 of these facilities yielded the following results: 106 (99%) infected residents presented with primary symptoms of vomiting and diarrhea; 84 (79%) presented with associated symptoms; median symptom duration was 4 days; and approximately 70% of infected residents were diagnosed on the basis of symptom progression alone. Isolation circumstances varied between facilities. Approximately 75% of infected residents received treatment, most commonly infusions and antibiotics. The median antibiotic cost was about twice the all-drug cost.

Almost all of the infected residents presented with vomiting and diarrhea, and approximately 80% presented with associated symptoms, including decreased appetite, fever of 37 °C or greater, and general malaise, over a median symptom duration of 4 days. In a study of 7 LTCFs in the United States where outbreaks of NG occurred between 2009 and 2013, 40 of the 62 patients were aged 70 years or older, all of the infected residents aged 70 years or older presented with vomiting and diarrhea, and median symptom duration was 4 days [20]. Up to this point, no studies in Japan or other Asian countries have investigated the clinical course of NG in multiple facilities for elderly persons based on medical records, making the present study the first of its kind. The clinical course of NG in facilities in this study was found to be nearly consistent with that previously reported for facilities in the United States.

Approximately 75% of infected residents were administered some type of treatment, with approximately 60% receiving infusion intravenously and approximately 30% receiving antibiotics. This study showed the actual state of treatment of NG patients in LTCFs, which had been unknown. Measures to prevent or control norovirus infection include hand washing, isolation of infected persons, and environmental disinfection; the recommended treatment is fluid when needed based on symptoms [4, 21]. More than half of infected residents in this study received intravenous fluid therapy, suggesting compliance with the MHLW manual. In contrast, the reasons for administration of antibiotics are not clear. According to previous studies, facilities for the elderly account for 47 to 79% of all antibiotic use in facilities every year, with 70% being administered to patients suffering from urinary tract and respiratory infections [22, 23]. It has been reported that 16 to 40% of antibiotic use in facilities is inappropriate [22, 24]. Of the 30 infected residents in this study who were administered antibiotics, 2 had symptoms besides vomiting and diarrhea associated with norovirus infection (1 patient was making abnormal respiratory sounds, and 1 patient had pneumonia) that might require treatment with antibiotics. Given that antibiotics are not expected to affect norovirus, the administration of antibiotics to the other 28 patients may have been inappropriate. Antibiotics should be administered cautiously to elderly individuals whose overall health status is in decline.

Approximately 70% of 107 infected residents were diagnosed on the basis of symptom progression. Methods for detecting norovirus include nucleic acid-based PCR methods and antigen-based immunoassay methods. The former methods are typically performed by government agencies or research entities to identify the causal agents of mass outbreaks of food poisoning or infection and require approximately 6 h to obtain results. Accordingly, antigen-based immunoassay methods, which are easy to perform and yield results in approximately 20 min or less, are more suitable for facilities [25]. However, immunoassays have a sensitivity of 92% and specificity of 98%, resulting in a high probability of false-negative results [25]. According to the cooperating facilities in this study, one set of immunoassays (10 tests) costs between USD 150 and 200, which is paid entirely by the facilities. It is for this reason that most of the infected residents were diagnosed on the basis of clinical presentation.

Although both the CDC guidelines and the MHLW manual recommend isolation of infected residents to prevent spread of norovirus infection, the actual circumstances of isolation differed between facilities [21]. Only about half of the infected residents in two facilities, which had large numbers of infected residents, were isolated. While the detailed reasons for this are not clear, there may have been a shortage of rooms or beds to use for isolation due to facility design or a shortage of staff to implement isolation. Placing infected and non-infected residents in the same room increases the risk of further infection. There is a need to determine why some of the infected residents were not isolated and to identify measures to resolve this issue.

The median total drug cost for the 81 patients who were administered some type of medication was USD 4.4, and the median drug cost per patient per day was USD 2.0. As far as we are aware, no previous studies have calculated the costs of drugs administered. The drug costs obtained in this study are not high and do not constitute a substantial economic burden on facilities. That said, while the costs of antibiotics are not high, given the possibility that the administration of antibiotics was inappropriate in the majority of cases, antibiotic use should be reviewed. NG outbreaks can have a large economic impact on facilities through restriction of new patient intake, disruption of work if staff become infected, and decreased public confidence in facilities due to harmful rumors [2]. Given these indirect costs, it is worthwhile to examine the potential financial and social losses that will be incurred if measures to prevent the outbreak and spread of NG are not implemented appropriately.

There are a number of limitations to this study. First, because there is no established diagnostic standard for NG, residents who were diagnosed by facilities to be infected with NG were designated “infected residents.” Doctors in facilities made NG diagnoses based on the presence of symptoms typical of NG, timing of incidence, incidence among other residents, staff reports of residents’ symptoms, and their own experience. Because there is a possibility that facilities treat all residents with NG-like symptoms as being infected as a precautionary measure to prevent further spread of infection within the facility, the number of infected residents may have been overestimated. Meanwhile, given the error based on the accuracy of diagnostic kits, the number of infected residents diagnosed using diagnostic kits may have been underestimated. The second limitation has to do with the fact that it was only possible to examine the medical records of infected residents in the 5 facilities that granted permission to do so. The 5 cooperating facilities had a mean capacity of 99 patients and a median average occupancy rate of 97%, making them typical examples of the 3533 geriatric intermediate care facilities in Japan (mean capacity of 91 patients, mean occupancy rate of 92%) [26]. However, we believe that the results of this study were obtained from a group of facilities that pay relatively greater attention to preventing and dealing with infection. Thus, caution must be exercised when generalizing these findings to other LTCFs. Third, with regard to additional expenses associated with the NG outbreaks at LTCFs, it was only possible to clarify the costs of drugs and other medical supplies directly used in treating the patients. NG outbreaks generate indirect costs associated with limited admission of new patients, staff sick leave and overtime, cleaning fees, and so on. The data on the duration of limited admissions and the numbers of patients admitted during these periods that could be obtained (median duration (range): 20 days (11 to 52 days); 0 to 2 patients) suggest that the financial impact on facilities was small. It was not possible to obtain data on whether staff were infected and took sick leave as a result of any of the outbreaks investigated in this study. No additional cleaning fees were incurred as a result of the NG outbreaks. This is because, in the facilities investigated, the cleaning was not outsourced to a third party but, rather, performed as part of staff duties, which is typical practice for LTFCs in Japan.

## Conclusion

The clinical course of almost all infected residents was typical for NG. Approximately 75% of the infected residents were administered some type of medication, most commonly infusions and antibiotics, with the highest cost being associated with antibiotics. Although the costs of the drugs were not very high, the appropriateness of different drug treatments should be examined.

## Supplementary information

**Additional file 1.** Questionnaires of Surveys I.

**Additional file 2.** Questionnaires of Surveys II.

## Data Availability

The data used in this study are not available and shared, since consent to provide them was not obtained from the cooperating facilities.

## Abbreviations

NG: Norovirus gastroenteritis
LTCFs: Long-term care facilities
MHLW: Ministry of Health, Labour and Welfare
JAGHSF: Japan Association of Geriatric Health Service Facilities

## References

[1] Rajagopalan S, Yoshikawa TT (2016). Norovirus infections in long-term care facilities. J Am Geriatr Sco.

[2] Belliot G, Lopman BA, Ambert-Balay K, Pothier P (2014). The burden of norovirus gastroenteritis: an important foodborne and healthcare-related infection. Clin Microbiol Infect.

[3] Hall AJ, Lopman BA, Payne DC, Patel MM, Gastanaduy PA, Vinje J, Parashar UD (2013). Norovirus disease in the United States. Emerg Infect Dis.

[4] Ministry of Health, Labour and Welfare. Portal site of Q&A of norovirus. 2016. http://www.mhlw.go.jp/file/06-Seisakujouhou-11130500-Shokuhinanzenbu/0000129187.pdf. Accessed 5 July 2019. (in Japanese).

[5] National Institute of Infectious Disease. Portal site of place of norovirus outbreak 2011/11–2016/17. 2017. http://www.nih.go.jp/niid/images/iasr/rapid/noro/160920/norosui3_170126.gif. Accessed 5 July 2019. (in Japanese).

[6] Wakisaka H, Shimizu N (2014). Questionnaire survey on current status of measures against infectious diseases at elderly care facilities in prefecture a. Jpn J Infect Prev Control.

[7] Takushima H, Yamamoto H, Tokuzumi K, Moritsuka M (2013). Infection control in long-term care facilities of present situation-results from an investigation of managers at long-term care facilities. J Health Sci.

[8] Lopman BA, Reacher MH, Vipond IB (2004). Epidemiology and cost of nosocomial gastroenteritis, Avon, England, 2002-2003. Emerg Infecti Dis.

[9] Fretz R, Schmid D, Jelovcan S (2009). An outbreak of norovirus gastroenteritis in an Austrian hospital, winter 2006-2007. Wien Klin Wochenschr.

[10] Piednoir E, Borderan GC, Borgey F (2010). Direct costs associated with a hospital-acquired outbreak of rotaviral gastroenteritis infection in a long term care institution. J Hosp Infect..

[11] Danial J, Cepeda JA, Cameron F, Cloy K, Wishart D, Templeton KE (2011). Epidemiology and costs associated with norovirus outbreaks in NHS Lothian, Scotland 2007–2009. J Hosp Infect..

[12] Robilotti E, Deresinski S, Pinsky BA (2015). Norovirus. Clin Microbiol Rev.

[13] Goller JL, Dimitriadis A, Tan A, Kelly H, Marshall JA (2004). Long-term features of norovirus gastroenteritis in the elderly. J Hosp Infect.

[14] Vega E, Barclay L, Gregoricus N, Shirley SH, Lee D, Vinjé J (2014). Genotypic and epidemiologic trends of norovirus outbreaks in the United States, 2009 to 2013. J Clin Microbiol.

[15] Fujiki S, Ishizaki T, Nakayama T (2017). Variations in status of preparation of personal protective equipment for preventing norovirus gastroenteritis in long-term care facilities for the elderly. J Evalu Clinical Pract.

[16] Ishizaki T, Kobayashi Y, Tamiya N (1998). The role of geriatric intermediate care facilities in long-term care for the elderly in Japan. Health Policy.

[17] Campbell JC, Ikegami N (2000). Long-term care insurance comes to Japan. Health Aff (Millwood).

[18] Campbell JC, Ikegami N, Gibson MJ (2010). Lessons from public long-term care insurance in Germany and Japan. Health Aff (Millwood)..

[19] Miquel Porta. A Dictionary of Epidemiology. 5th ed: Oxford University Press; 2008.

[20] Costantini VP, Cooper EM, Hardaker HL (2016). Epidemiologic, virologic, and host genetic factors of norovirus outbreaks in long-term care facilities. Clin Infect Dis.

[21] Division of Diseases, National Center for Immunization and Respiratory Disease, Centers for Disease Control and Prevention.Updated norovirus outbreak management and disease prevention guideline. Morbidity and Mortality Weekly Report Recommendation and Reports. 2011;60;RR-3:1–18. 21368741

[22] Stuart RL, Wilson J, Bellaard-Smith E (2012). Antibiotic use and misuse in residential aged care facilities. Intern Med J.

[23] Van Buul LW, van der Steen JT, Veenhuizen RB (2012). Antibiotic use and resistance in long term care facilities. J Am Med Dir Assoc.

[24] Sloane PD, Zimmerman S, Brown LC, Ives TJ, Walsh JF (2002). Inappropriate medication prescribing in residential care/assisted living facilities. J Am Geriatr Soc.

[25] Tomoyuki T (2012). Norovirus antigen detection Immunochromatography (IC) kit. J Modern Med.

[26] Ministry of Health, Labour and Welfare. Portal site of Survey of Institutions and Establishments for Long-term Care, 2010. http://www.mhlw.go.jp/toukei/saikin/hw/kaigo/service10/dl/kekka-gaiyou_03.pdf. Accessed 5 July, 2019. (in Japanese).

