# Supplementary material for: Clinical pictures, treatments, and resource use of norovirus gastroenteritis in long-term care facilities: a survey with a chart review in Japan
Source: BMC Geriatr. 2020 Apr 21;20:148. doi: 10.1186/s12877-020-01549-0 (PMC7171776; doi:10.1186/s12877-020-01549-0)
Supplement: Supplementary file 1 — Additional file 1. Questionnaires of Surveys I. [file 12877_2020_1549_MOESM1_ESM.docx]

**Additional file 1: Questionnaires of Surveys I**

1. Did your facility have any residence (except short-stay) who had norovirus gastroenteritis from 2009 October to 2010 March and from 2010 October to 2011 March? If your facility had such patients, please indicate the total number of patients your facility had.

1) From 2009 October to 2010 March

(1) had patients →　total number of patients ( ) →　Go to 2

(2) had no patients → Go to 4

2) From 2010 October to 2011 March

(1) had patients →　total number of patients ( ) →　Go to 2

(2) had no patients → Go to 4

2. Did you restrict admission of new residences to your facility from 2009 October to 2010 March and from 2010 October to 2011 March?

1) From 2009 October to 2010 March

(1) Yes → Go to 3

(2) No → Go to 4

2) From 2010 October to 2011 March

(1) Yes → Go to 3

(2) No → Go to 4

3. From 2009 October to 2010 March and from 2010 October to 2011 March, how long did you restrict new admissions to the facility and how many new residences were declined? Please fill in this form as in the example.

| **Restricted period (y/m/d - y/m/d)** | **Number of admissions declined (persons)** |
| --- | --- |
| **Ex) 2010/01/08 -2010/ 01/15** | **2** |
|  |  |
|  |  |

4. When your facility did have patients with symptom similar to norovirus gastroenteritis, what were the diagnostic criteria of norovirus gastroenteritis in your facility?

(1) It was diagnosed only by symptoms without using a diagnosis kit.

(2) It was diagnosed using a diagnosis kit.

5. Did you use a diagnosis kit during each period? When you did use a diagnostic kit, please fill in the number of diagnosis kits.

1) From 2009 October to 2010 March

(1) Yes →　　number of kits ( )

(2) No

2) From 2010 October to 2011 March

(1) Yes → number of kits ( )

(2) No

6. What was the unit price of the diagnosis kits used in your facility?

1) From 2009 October to 2010 March ( ) yen

2) From 2010 October to 2011 March ( ) yen

7. At the two following times, did you perform the prophylaxis at the time when there were no residents with infectious gastroenteritis during an outbreak?

1) From 2009 October to 2010 March

(1) Yes

(2) No

2) From 2010 October to 2011 March

(1) Yes

(2) No

8. At the two following times, how many patients did you have with each outcome?

1) From 2009 October to 2010 March

| **Outcome** | **Number of patients (persons)** |
| --- | --- |
| **Recovered in facility** |  |
| **Died in facility** |  |
| **Transferred to hospital** |  |

2) From 2010 October to 2011 March

| **Outcome** | **Number of patients (persons)** |
| --- | --- |
| **Recovered in facility** |  |
| **Died in facility** |  |
| **Transferred to hospital** |  |

9. Please fill in this form about basic information of your facility

・Mean occupancy rate ( ) %

・Mean of capacity ( ) persons

10. If possible, would you participate in the survey about 2011(from 2011 October to 2012 March) with the same content as this survey?

(1) Yes

(2) No

11. This is a question for facilities that had one or more patients with norovirus gastroenteritis from 2009 October to 2010 March and/or from 2010 October to 2011 March. Can the researcher with a nursing license perform a chart review of patients with norovirus gastroenteritis? The contents of the chart review are as follows.

・patients’ characteristics : sex, age, level of ADL, nursing care level, and clinical history

・symptoms and symptom duration

・use or non-use of diagnostic kit

・isolation circumstances, and isolation period

・treatment during the symptomatic period : name of medications, daily dose, and number of days administered for each medicine

・outcome

(1) Yes

(2) No

The questionnaire is finished.

Thank you very much for your cooperation during this busy period.
